# Case Study: HLA incompatible platelet infusion to allow emergency salvage HCT in a patient with primary graft failure due to donor specific HLA antibody

**DOI:** 10.3389/fimmu.2026.1752791

**Published:** 2026-02-16

**Authors:** Louise Goddard, Jonathan Moses, Gerard Joseph Chu, Robert P. Carroll

**Affiliations:** Australian Redcross Lifeblood, NSW Transplantation and Immunogenetics, Sydney, NSW, Australia

**Keywords:** donor specific antibody, infusion, platelet, refractoriness, stem cell transplantation

## Abstract

A patient with myelodysplasia received a haploidentical hematopoietic stem cell transplant (HCT). Pre-transplant HLA antibody screening showed no detectable donor-specific antibodies (DSAs). Post-transplant, the patient developed an anti-HLA-A2 antibody, likely due to a memory B-cell response. This immune response led to platelet transfusion refractoriness and graft failure. The patient engrafted following a second stem cell infusion from the same haploidentical donor, using both HLA compatible platelets, to support initial platelet transfusion refractoriness (PTR) following primary stem cell infusion, and HLA-A*02 expressing platelets, to adsorb the circulating anti-A2 prior to the re-infusion.

## 1 Introduction

HCTs are used to treat conditions in which the bone marrow is unable to produce healthy blood cells (1). The ideal HCT donor is an HLA matched [sibling or matched unrelated (MUD)] with the patient (HLA-A, -B, -C, -DRB1, -DQB1, -DPB1). Unfortunately, not all patients will have an ideal donor. There are ongoing efforts to identify alternative donor options, including haploidentical related donors. The challenge of HLA mismatched transplants is controlling host versus graft immune responses to allow effective engraftment (1, 2). Anti-HLA DSA are an important cause of engraftment failure and may negatively impact survival outcomes of patients receiving allogeneic HCT using an HLA-mismatched donor (3). The development of HLA antibodies can result from a memory response of the recipient, or *de novo* antibodies produced by the recipient against the HLA-mismatched cells (4–6).

It is widely acknowledged that a combination of desensitization strategies may be required to treat a HCT patient with anti-HLA DSA. Treatments currently being used are a combination of protocols that include the removal, neutralization, and inhibition of antibodies, as well as blocking of antibody production to prevent activation of the complement cascade (1, 7–9).

HLA alloantibodies against the donor HLA Class I antigens expressed at the cell surface of platelets results in destruction and removal of the platelets from circulation, leading to refractoriness and graft failure (1, 3, 10). Leucocyte and platelet count after HCT serve as early indicators for the success of transplantation with the time to engraftment of platelets appearing to be a predictor of the probability of complications in transplant recipients (11). Patients with platelet refractoriness can be supported through transfusion of HLA-compatible platelets, that is, excluding donors who have the HLA antigen for which the patient has antibodies to (12, 13).

## 2 Case description

This patient case study investigates HLA matched and HLA non-matched platelet transfusion support options for a haploidentical HCT recipient. The 40-year-old female patient with myelodysplastic syndrome developed strong HLA DSA from an apparent post-transplant memory response with subsequent graft failure. The patient had two prior pregnancies and a history of transfusion (both platelet and blood).

The patient presented in January 2023 for a HCT work-up. Initial HLA typing using AllType FASTPlex™ next generation sequencing (NGS) (One Lambda™) and verification typing using LABType™ SSO (One Lambda™) technology were performed (**Table 1**). HLA Class I and Class II antibody screening were performed using LABScreen™ Single Antigen (One Lambda™) bead technology (SAB). Serum was treated using EDTA to mitigate complement inhibition. A mean fluorescence intensity (MFI) cutoff of 500 is deemed positive. A anti-A25 (MFI 1700) was detected. No Class II antibodies were detected.

**Table 1:** A family search was performed, and two siblings were initially assessed as potential donors.

Family Member	HLA Typing								
	A	B	C	DRB1	DRB345	DQB1	DQA1	DPB1	DPA1
Patient	24:02:01	46:01:01	1:02:01	09:01/09:31	DRB4*	3:03:02	3:02:01	5:01	2:02:02
					1:03:01				
	3:01:01	44:02:01	7:04:01	13:02:01	DRB3*	6:04:01	1:02:01	4:01:01	1:03:01
					3:01:01				
Donor 1 Brother	24:02:01	46:01:01	1:02:01	9:01:02	DRB4*	3:03:02	3:02:01	5:01:01	2:02:02
					1:03:01				
	2:01:01	7:02:01	7:02:01	15:01:01	DRB5*	6:02:01	1:02:01	4:01:01	1:03:01
					01:01/01:126				
Donor 2 Sister	24:02	46:01		9:01					
	2:01	7:02		15:01					

HLA typing using LABTypeTM SSO, One Lambda™, ThermoFisher Scientific, was performed. While neither sibling was HLA identical to the patient, they both potentially shared a haplotype with the patient. One sibling (brother) underwent further HLA typing by AllType FASTPlex™ NGS, OneLambda™, ThermoFisher Scientific, to confirm haploidentical status. Shared haplotype between the patient and preferred donor (brother) is highlighted.

Two siblings were tested by LABType™ SSO. HLA-A, -B and -DRB1 testing indicated neither sibling was HLA identical to the patient, but they both potentially shared a haplotype with the patient (**Table 1**). DSA were not detected against either sibling.

Concurrently, the patient, of North-West European ancestry, was also considered for a MUD transplant. Of the patient’s inherited haplotypes, one was common in Asia/Pacific Island populations, the other was an uncommon haplotype in the Caucasian population. A search of international registries failed to find a potential unrelated donor who was more than a 7/10 HLA-A, -B, -C, -DRB1, -DQB1 match.

In October 2023, the transplant unit decided to progress a haploidentical HCT with the patient’s brother. Verification typing using AllType FASTPlex™ NGS (One Lambda™) was performed (**Table 1**). SAB was performed on sample dated 04 Oct 2023. The patient had an anti-A25 (MFI 582) and no HLA Class II antibodies. No DSA to the intended haploidentical donor were identified.

The patient underwent myeloablative conditioning and a HCT (peripheral blood stem cell (PBSC)) on 07 Nov 2023. Following the HCT, the patient developed bleeding complications and had a platelet count of 10x10^9^/L on 12 Nov 2023. After transfusion, there was no increment in platelet counts [11x10^9^/L (1 hour) and 9x10^9^/L (24 hours)] and ongoing platelet transfusion support was provided.

SAB screening on post-HCT sample, 16 Nov 2023, showed development of strong DSA anti-A2 (MFI 23926). Epitope analysis identified two antibody verified eplets, 62GE and 144TKH. As per EBMT guidelines (1), the sample was further tested using C1qScreen™ to identify the presence of complement binding DSA, anti-A2 (MFI 8960) were detected. Pre-transplant sample (04 Oct 2023) SAB results were retested, confirming original results, with no A2 epitope present below the cutoff.

Despite ongoing HLA-compatible platelet support, the patient continued to be refractory. No human platelet antigen (HPA) alloantibodies or autoantibodies were detected in the platelet immunofluorescence test and monoclonal antibody immobilization of platelet antigen assays (data not shown), excluding HPA alloantibodies as a source of refractoriness (14).

HLA antibody desensitization therapies including plasma exchange, intravenous immunoglobulin (IVIg), bortezomib and rituximab were initiated to support the patient. The patient progressed to a second HCT. The potential donor options and condition of the patient were key factors in determining options. The parents were evaluated as potential donors (data not shown). The patient’s offspring were not considered as potential donors due to their age (both <5 years old at the time).

The patient underwent reduced intensity conditioning (RIC) for a second HCT (PBSC) from the same haploidentical donor (A*02 mismatched) on 13 Dec 2023. The patient was transfused with three units of HLA-A*02 typed platelets prior to the HCT, to adsorb the anti-A2. The patients anti-A2 MFI was 20479, reduced from MFI 27953 on sample dated 12 Dec 2023. The anti-A2 was MFI 9237 on a sample dated 14 Dec 2023, < one day post second stem cell infusion.

**Figure 1:**
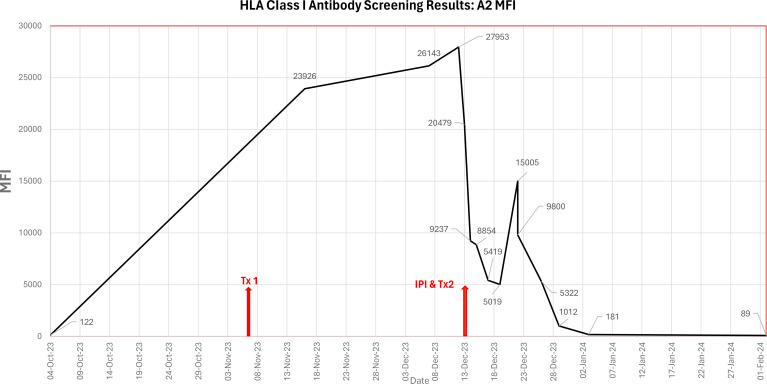
Graphical representation of the MFI of the anti-HLA A2 antibody in the patient’s serum from pre-transplant work-up until confirmed engraftment, including primary HCT (Tx1 07/Nov/2023), incompatible platelet infusion (IPI) and secondary HCT (Tx2 13/Dec/2023).

Ongoing antibody screening of the patient showed a further reduction in MFI for sample dated 17 Dec 2023: anti-A2 (MFI 5419), however, the anti-A2 DSA rebounded to MFI 15005 nine days post second HCT (22 Dec 2023). Ongoing platelet transfusion support for the patient was provided, excluding donors typed as HLA A*02. The patient also received plasma exchange therapy. Ongoing SAB screening of the patient was performed to confirm the presence of anti-A2 and to assess the strength (MFI). See **Table 2** and **Figure 1** for summary.

**Table 2:** MFI levels of anti-A2 in patient’s serum over course of treatment, using LABScreen™ Single Antigen and C1qScreen™ Assay, One Lambda™, ThermoFisher Scientific.

Sample date	Time of collection (if applicable)	Commentary	SAB A2 MFI	C1q A2 MFI
04-Mar-23	Unknown	Initial patient screen, transplant work-up	77	Not tested
04-Oct-23	09:40am	Pre-transplant screen	122	Not tested
07-Nov-23		Primary stem cell infusion		
16-Nov-23	08:04am	Post first transplant screen	23926	8960
07-Dec-23	06:25am		26143	22036
12-Dec-23	06:10am	Plasma exchange Primary graft failure confirmed	27953	23684
13-Dec-23		Incompatible A2 platelet support		
13-Dec-23	14:10pm	Pre second stem cell infusion	20479	18153
13-Dec-23		Second stem cell infusion		
14-Dec-23	06:00am	Post second transplant screen	9237	7158
15-Dec-23	18:55pm	Collected after plasma exchange	8854	64
17-Dec-23	13:20pm	Collected after plasma exchange	5419	7
19-Dec-23	15:30pm	Collected after plasma exchange	5019	Not tested
22-Dec-23	09:20am	Pre plasma exchange	15005	0
22-Dec-23	12:40pm	Collected after plasma exchange	9800	0
26-Dec-23	14:02pm	Collected after plasma exchange	5322	Not tested
29-Dec-23	12:30pm	Compatible platelet support	1012	0
03-Jan-24	06:30am	Compatible platelet support	181	Not tested
02-Feb-24	16:35pm	Compatible platelet support	89	Not tested

The patient’s antibody levels continued to be monitored by the laboratory and forty days post second HCT (02 Feb 2024), the patient’s SAB result showed no anti-A2 antibodies. Full engraftment was confirmed by the transplant unit on 13 Mar 2024.

## 3 Discussion

This is a case of a patient who developed strong DSA (anti-A2) following a stem cell infusion from a haploidentical donor, resulting in PTR and failure to engraft. A multimodal treatment strategy, including using A*02 platelets to adsorb circulating anti-A2 prior to reinfusion from the same haploidentical donor, resulted in successful engraftment.

Anti-HLA DSA are an important cause of engraftment failure and may negatively impact survival outcomes of patients receiving allogeneic HCT using an HLA-mismatched donor (3). The EBMT has guidelines for the detection and treatment of DSA during haploidentical HCT (1). Monitoring DSA levels before and after HCT could guide preemptive treatment when high levels persist after stem cell infusion. It is widely acknowledged that a combination of desensitization strategies may be required to treat a HCT patient with anti-HLA DSA, that is, there is no consensus protocol. The desensitization approach required can vary depending upon the class of HLA antibody, the intensity (MFI) of the antibody and its capacity to bind complement (7, 8). Treatments currently being used are a combination of protocols that include the removal, neutralization, and inhibition of antibodies, as well as blocking of antibody production to prevent activation of the complement cascade (1, 7–9). Higher MFI levels correlate with complement binding ability which may contribute to a higher chance of rejection in these patients (3, 15). The treatment strategies aim to decrease total antibody levels to allow successful engraftment of donor cells.

DSA can be removed by plasmapheresis or immunoadsorption noting that following plasmapheresis, antibody levels can rebound when donor stem cells are infused (13, 16). DSA can be neutralized using IVIg or with donor HLA antigens via platelet infusions for patients with HLA Class I antibodies (8, 9, 17). Strategies to inhibit antibody production include using monoclonal antibodies to CD20+ B lymphocytes (e.g. rituximab) and protease inhibitors (e.g. bortezomib) against antibody producing plasma cells, noting levels of already existing DSA will not be reduced with these strategies (1, 7, 9).

In several previously reported cases (7, 9, 16, 18–20), the presence of HLA DSA was known prior to transplantation. Spriewald et al. demonstrated that a patient with anti-A2 DSA showed a reduction in DSA intensity following mismatched platelet transfusion (seven units), with the patient successfully engrafting (19). Yoshihara et al. established that platelet transfusion from donors with DSA-corresponding HLA antigens on day -1 was particularly effective in reducing DSA levels on day 0, acknowledging the number of patients evaluated was not sufficient to draw solid conclusions (16). Yamashita et al. demonstrated reduction in DSA MFI levels following multiple platelet transfusions (twenty units) on the day of transplant, before infusion of donor cells. The recipient had rituximab (day -10) and IVIg (day -8 to -5). The effect of rituximab was modest and IVIg did not affect the titer of HLA antibodies at all, further supporting the platelet adsorption process as being integral to the reduction of DSA observed (9). Zhang et al., in a limited patient cohort, concluded that donor platelet infusion is a treatment option to neutralize anti-HLA Class I antibodies without increasing the risk of graft versus host disease (7). In a further case, Yamashita et al. demonstrated the effectiveness of mismatched platelet-based desensitization for anti-C is limited, with the expression of HLA-C on platelets a contributing factor (20). In these cases, awareness of the presence of HLA DSA prior to transplant allowed the units to therapeutically treat the recipient to eliminate or reduce the antibody level prior to transplantation, to better aid a successful engraftment.

The concept of removing HLA Class I antibodies by using pooled platelets as the *in-vivo* adsorbing material is well established (1, 4, 9, 17). Further, other groups focused on blocking the DSA with HLA antigens from the donor using a buffy coat (white blood cells) prepared from the donor cells (using HLA antigen of the donor) (1, 9, 15). The benefit of using a buffy coat over platelets to adsorb antibody is the ability to bind both HLA Class I and Class II antibody (15).

In the case presented here, there was no circulating DSA present pre-transplant to potentially predict the immune response and allow the transplant unit to consider treatment options prior to HCT. Prior to the primary HCT, the patient appeared to have low alloantibody sensitization, their sensitization history included pregnancy and transfusion. The patient’s rapid antibody response post-transplant is indicative of immune mediated memory B-cell involvement (5) and suggests prior exposure to the HLA-A*02 antigen and cryptic sensitization. In a study aiming to validate the role of DSAs as a risk factor in graft failure, Lima et al. speculated that a patient who failed to engraft could have cryptic HLA sensitization, with the observed post-transplant *de novo* DSA associated with a memory response (3).

There have been several reported cases of *de novo* DSA development resulting in failure to engraft in patients without circulating alloantibody in pre-HCT samples (21, 22). Yabe et al. reported a case of graft rejection associated with *de novo* anti-HLA DQB/DQA antibody development, where they confirmed anti-HLA did not transfer from blood product or gamma globulin, concluding that the DSA in the patient generated from residual host cells (21). While Hefazi et al. reported a single case study of *de novo* anti-HLA DPB1 resulting in graft failure following a RIC protocol (22). Both cases highlighting the importance of testing for DSA post HCT if unexplained pancytopenia develops.

Despite advances in technology, donor-specific HLA-specific memory B-cells may be present in some recipients with no detectable alloantibody sensitization. Assay options to assess memory B-cells responses are available but data has shown they have limitations in providing the prevalence, frequency, specificity and persistence of HLA antibody (3, 6, 23). In Australia, HLA compatible platelets for patients who are planned to undergo a haploidentical HCT, where development of HLA antibodies could result in an adverse transplantation outcome, can be provided prior to the transplant until the day of their stem cell infusion (day 0), irrespective of their HLA antibody status. Currently, no guidelines in Australia exist for non-compatible platelet infusion for desensitization purposes (24).

Before the second stem cell infusion from the same haploidentical donor, the patient received A*02 typed platelets. This approach aimed to adsorb circulating anti-A2, reducing levels before re-infusion of the donor HLA-A*02 stem cells. The intent of the treatment was to mitigate antibody-mediated barriers to engraftment. However, platelet adsorption alone cannot fully explain the subsequent engraftment, as the patient also underwent desensitization therapy with IVIg, bortezomib, and rituximab. Consequently, the relative contribution of each intervention to the antibody reduction remains uncertain. Notably, the MFI of the anti-A2 decreased from 27953 to 20479 immediately prior to the second infusion, which is consistent with antigen adsorption.

Further research into HLA DSA treatment strategies for HCT is required, including the effectiveness of platelet adsorption to reduce HLA Class I DSA. Most of the published data regarding HCT outcome in the presence of anti-HLA DSA is limited to individual case reports and small studies and not multi-center or controlled trials.

## 4 Conclusion

This is a case of a patient who was able to be supported through to engraftment, from a second stem cell infusion, using both HLA compatible (to support initial platelet transfusion refractoriness following primary stem cell infusion) and incompatible platelets (to adsorb the anti-A2 antibodies prior to a second stem cell infusion). The case highlights the clinical utility of targeted antibody adsorption as part of a multimodal treatment strategy to overcome DSA mediated engraftment failure, underscoring its potential role in expanding treatment options for haploidentical stem cell transplantation where immunologic barriers remain a challenge. Further, research into the effectiveness of HLA incompatible platelets for desensitization in sensitized HCT patients is required through clinical trials.

## Data Availability

The original contributions presented in the study are included in the article/supplementary material. Further inquiries can be directed to the corresponding author.

## References

[1] Kongtim P Vittayawacharin P Zou J Srour S Shaffer B Shapiro RM . ASTCT consensus recommendations on testing and treatment of patients with donor-specific anti-HLA antibodies. Transplant Cell Ther. (2024) 30:1139–54. doi: 10.1016/j.jtct.2024.09.005, PMID: 39260570 PMC12637036

[2] Little AM . HLA antibodies in haematopoietic stem cell transplantation. HLA. (2019) 94 Suppl 2:21–4. doi: 10.1111/tan.13741, PMID: 31674146

[3] Lima ACM Getz J do Amaral GB Loth G Funke VAM Nabhan SK . Donor-specific HLA antibodies are associated with graft failure and delayed hematologic recovery after unrelated donor hematopoietic cell transplantation. Transplant Cell Ther. (2023) 29:493. doi: 10.1016/j.jtct.2023.05.014, PMID: 37220839

[4] Brand A Doxiadis IN Roelen DL . On the role of HLA antibodies in hematopoietic stem cell transplantation. Tissue Antigens. (2013) 81:1–11. doi: 10.1111/tan.12040, PMID: 23216286

[5] Chong AS Sciammas R . Memory B cells in transplantation. Transplantation. (2015) 99:21–8. doi: 10.1097/TP.0000000000000545, PMID: 25525921 PMC4273865

[6] Chong AS Ansari MJ . Heterogeneity of memory B cells. Am J Transpl. (2018) 18:779–84. doi: 10.1111/ajt.14669, PMID: 29359404 PMC5962275

[7] Zhang R He Y Yang D Jiang E Ma Q Pang A . Combination treatment of rituximab and donor platelets infusion to reduce donor-specific anti-HLA antibodies for stem cells engraftment in haploidentical transplantation. J Clin Lab Anal. (2020) 34. doi: 10.1002/jcla.23261, PMID: 32112480 PMC7370703

[8] Bailén R Vicario JL Solán L Sánchez-Vadillo I Herrera P Calbacho M . Management of donor-specific antibodies in haploidentical transplant: multicenter experience from the madrid group of hematopoietic transplant. Front Immunol. (2021) 12:674658. doi: 10.3389/fimmu.2021.674658, PMID: 34093576 PMC8170127

[9] Yamashita T Ikegame K Kojima H Tanaka H Kaida K Inoue T . Effective desensitization of donor-specific HLA antibodies using platelet transfusion bearing targeted HLA in a case of HLA-mismatched allogeneic stem cell transplantation. Bone Marrow Transpl. (2017) 52:794–6. doi: 10.1038/bmt.2017.10, PMID: 28165448

[10] Couvidou A Rojas-Jiménez G Dupuis A Maître B . Anti-HLA Class I alloantibodies in platelet transfusion refractoriness: From mechanisms and determinants to therapeutic prospects. Front Immunol. (2023) 14:1125367. doi: 10.3389/fimmu.2023.1125367, PMID: 36845153 PMC9947338

[11] Doescher A Casper J Kraemer D Kapels HH Petershofen EK Müller TH . Platelet engraftment after allogenic stem cell transplantation is monitored by digital polymerase chain reaction without interference by platelet support. Exp Hematol. (2018) 68:21–9. doi: 10.1016/j.exphem.2018.08.007, PMID: 30195456

[12] Pavenski K Freedman J Semple JW . HLA alloimmunization against platelet transfusions: pathophysiology, significance, prevention and management. Tissue Antigens. (2012) 79:237–45. doi: 10.1111/j.1399-0039.2012.01852.x, PMID: 22385314

[13] Hod E Schwartz J . Platelet transfusion refractoriness. Br J Haematol. (2008) 142:348–60. doi: 10.1111/j.1365-2141.2008.07189.x, PMID: 18510692

[14] NHSBT . Guidelines for the Management Platelet Transfusion Refractoriness (INFORMATION DOCUMENT 139/2, Effective from 28/07/2021).

[15] Ciurea SO Thall PF Milton DR Barnes TH Kongtim P Carmazzi Y . Complement-binding donor-specific anti-HLA antibodies and risk of primary graft failure in hematopoietic stem cell transplantation. Biol Blood Marrow Transpl. (2015) 21:1392–8. doi: 10.1016/j.bbmt.2015.05.001, PMID: 25985919 PMC4506716

[16] Yoshihara S Maruya E Taniguchi K Kaida K Kato R Inoue T . Risk and prevention of graft failure in patients with preexisting donor-specific HLA antibodies undergoing unmanipulated haploidentical SCT. Bone Marrow Transpl. (2012) 47:508–15. doi: 10.1038/bmt.2011.131, PMID: 21691261

[17] Jung J Barron C . Elimination of HLA antibodies by platelet adsorption. Immunohematology. (2020) 36:1–3. doi: 10.21307/immunohematology-2020-034, PMID: 32324037

[18] Misra MK Xin JJ Brown NK Weidner JG Upchurch RL Bishop MR . Effective desensitization for a strong donor-specific HLA antibody in a case of HLA-mismatched allogeneic hematopoietic cell transplantation. HLA. (2019) 94:307–11. doi: 10.1111/tan.13627, PMID: 31314169

[19] Spriewald BM Bach C Zingsem J Strobel J Winkler J Mackensen A . Depletion of donor-specific anti-HLA A2 alloantibodies in a hematopoietic cell transplant recipient using directed mismatched platelet transfusions. Bone Marrow Transplant. (2018) 53:791–4. doi: 10.1038/s41409-018-0220-7, PMID: 29795430 PMC6006140

[20] Yamashita T Ikegame K Ito F Kobayashi T Nara M Fujishima N . Effect of low platelet HLA-C expression on donor-specific antibody depletion following platelet transfusion from a corresponding HLA donor. Bone Marrow Transplant. (2019) 54:1713–6. doi: 10.1038/s41409-019-0482-8, PMID: 30824816

[21] Yabe H Morimoto T Takakura H Okuya M Ikegaya R Kato S . Post-transplantation-emerging anti-HLA DQA1/DQB1 antibody possibly responsible for graft rejection after myeloablative-unrelated marrow grafting. Bone Marrow Transpl. (2016) 51:601–3. doi: 10.1038/bmt.2015.292, PMID: 26642341

[22] Hefazi M Hogan WJ Wakefield LL Gandhi MJ . The association of *de novo* anti-HLA-DPB1 donor-specific antibody formation and primary graft failure after allogeneic hematopoietic cell transplantation. Hum Immunol. (2018) 79:861–4. doi: 10.1016/j.humimm.2018.08.009, PMID: 30142360

[23] Nellore A Killian JT Jr Porrett PM . Memory B cells in pregnancy sensitization. Front Immunol. (2021) 12:688987. doi: 10.3389/fimmu.2021.688987, PMID: 34276679 PMC8278195

[24] ANZSBT Guidelines . Guidelines for Transfusion and Immunohaematology Laboratory Practice, 2nd Edition. Sydney, Australia: Australian and New Zealand Society of Blood Transfusion (ANZSBT) Ltd (2025).

